# *Aporocotyle mariachristinae* n. sp., and *A. ymakara* Villalba & Fernández, 1986 (Digenea: Aporocotylidae) of the pink cusk-eel, *Genypterus blacodes* (Ophidiiformes: Ophidiidae) from Patagonia, Argentina

**DOI:** 10.1051/parasite/2012194319

**Published:** 2012-11-15

**Authors:** J.S. Hernández-Orts, G. Alama-Bermejo, J.M. Carrillo, N.A. García, E.A. Crespo, J.A. Raga, F.E. Montero

**Affiliations:** 1 Cavanilles Institute of Biodiversity and Evolutionary Biology, Science Park, University of Valencia, C/ Catedrático José Beltrán 2 46980 Paterna, Valencia Spain; 2 Laboratory of Fish Protistology, Institute of Parasitology, Biology Centre, ASCR, Branisovska 31 370 05 Ceske Budejovice Czech Republic; 3 Department of Coastal Sciences, University of Southern Mississippi 703 East Beach Drive 39564 Ocean Springs, Mississippi USA; 4 Marine Mammal Laboratory, National Patagonic Center, CONICET and University of Patagonia Boulevard Brown 2915 (9120) Puerto Madryn, Chubut Argentina

**Keywords:** Aporocotylidae, *Aporocotyle*, *A. mariachristinae* n. sp., *A. ymakara*, *Genypterus blacodes*, Ophidiidae, Patagonia, Argentina, rDNA sequences, Aporocotylidae, *Aporocotyle*, *A. mariachristinae* n. sp., *A. ymakara*, *Genypterus blacodes*, Ophidiidae, Patagonie, Argentine, séquençage, ADN ribosomique

## Abstract

*Aporocotyle mariachristinae* n. sp. and *A. ymakara* Villalba & Fernández, 1986 were collected from the bulbus arteriosus and ventral aorta of pink cusk-eels, *Genypterus blacodes* (Forster, 1801) from Patagonia, Argentina. *A. mariachristinae* n. sp. can be distinguished from all the species of *Aporocotyle* by the asymmetrical extension of posterior caeca (right posterior caecum longer, terminating at the area between mid-level of ovary and posterior body end; left posterior caecum shorter, terminating at the area between mid-level of cirrus sac and posterior to reproductive organs), the distribution of spines along the ventro-lateral body margins and the number of testes. The new species clearly differs from *A. ymakara*, from the same host species, in the esophagus / body length ratio, the absence of distal loops at caeca, the anterior caeca / posterior caeca length ratio, and the number of testes. Additionally, in *A. ymakara* the left posterior caecum may be longer than right posterior caecum, while in the new species left posterior caecum is always shorter. The specimen of *A. ymakara* collected from Argentina is also described. We also provide observations of the distribution of spines in different species of *Aporocotyle*, including new specimens of *A. argentinensis* Smith, 1969 from *Merluccius hubbsi* Marini, 1933. Molecular sequence data obtained from partial 18S and 28S rDNA regions were compared between the new species and other two species of *Aporocotyle* (*A. argentinensis* and *A. spinosicanalis* Williams, 1958). This is a new locality record for *A. ymakara*, extending the known geographical distribution for this species from Chile to Argentina, and the first report of two species of *Aporocotyle* in the same host species and locality.

## Abstract (fr)

Les espèces *Aporocotyle mariachristinae* n. sp. et *A. ymakara* Villalba & Fernández, 1986 ont été prélevées sur le bulbe artériel et l’aorte ventrale d’abèches roses *Genypterus blacodes* (Forster, 1801) de Patagonie, Argentine. *A. mariachristinae* n. sp. se distingue de toutes les autres espèces d’*Aporocotyle* par la prolongation asymétrique de son caecum postérieur (le caecum postérieur droit est plus long et s’achève à un endroit situé à mi-hauteur entre l’ovaire et l’extrémité postérieure du corps ; le caecum postérieur gauche est moins long et s’achève à un endroit situé à mi-hauteur entre le cirrus, le postérieur et les organes reproducteurs), par la distribution des épines qui longent les parties ventro-latérales du corps, et encore par le nombre de testicules. Cette nouvelle espèce se différencie clairement de l’espèce *A. ymakara*, qui parasite la même espèce, par la proportion entre la longueur de l’oesophage et celle du corps, par l’absence de boucle distale au niveau du caecum, par la proportion entre la longueur du caecum antérieur et celle du caecum postérieur, ainsi que par son nombre de testicules. De plus, on remarque que chez *A. ymakara*, le caecum postérieur gauche peut être plus long que le caecum postérieur droit, alors que chez la nouvelle espèce, le caecum postérieur gauche est toujours plus court. Le spécimen d’*A. ymakara* recueilli en Argentine fait aussi l’objet d’une description. Nous faisons aussivpart de nos observations concernant la distribution des épines chez les différentes espèces d’*Aporocotyle*, y compris chez de nouveaux spécimens d’*A. argentinensis* Smith, 1969 prélevés sur le *Merluccius hubbsi* Marini, 1933. Nous avons comparé les séquences moléculaires obtenues à partir de régions particulières d’ADN ribosomique 18S et 28S chez la nouvelle espèce et chez deux autres espèces d’*Aporocotyle* (*A. argentinensis* et *A. spinosicanalis* Williams, 1958). *A. ymakara* fait donc l’objet d’une nouvelle répartition géographique qui s’étend désormais du Chili à l’Argentine. C’est la première fois que deux espèces d’*Aporocotyle* sont recensées sur une même espèce-hôte et dans une même zone géographique.

## Introduction

*Aporocotyle* Odhner, 1900 is the type genus of the family Aporocotylidae (Bullard *et al.*, 2009). Species of *Aporocotyle* infect the heart, bulbus arteriosus, ventral aorta and branchial vessels, and other blood vessels of fishes of five teleost orders: Gadiformes, Ophidiiformes, Perciformes, Pleuronectiformes and Scorpaeniformes (Yamaguti, 1934, 1970; Williams, 1958; Smith, 1967, 1969; Ichihara, 1970; Holmes, 1971; Parukhin & Tkachuk, 1980; Thulin, 1980; Fernández & Durán, 1985; Parukhin, 1985; Villalba & Fernández, 1986a, b; Tantaleán & Martínez, 1990). From the order Ophidiiformes, five species of *Aporocotyle* were originally described only in fishes of the genus *Genypterus*: *A. smithi* Parukhin & Tkachuk, 1980, from *G. capensis* (Smith, 1847); *A. keli* Villalba & Fernández, 1986, from *G. chilensis* (Guichenot, 1848); *A. kuri*Villalba & Fernández, 1986, from *G. maculatus* (Tschudi, 1846); *A. ymakara* Villalba & Fernández, 1986, from *G. blacodes* (Forster, 1801); and *A. garciai*Tantaleán & Martínez, 1990 from *Genypterus* sp. (Parukhin & Tkachuk, 1980; Villalba & Fernández, 1986b; Tantaleán & Martínez, 1990). More recently, Kamegai *et al.* (2002) reported *A. garciai* in a different host species belonging to a different ophidiid genus, *Hoplobrotula armata* (Temmink & Schlegel, 1846), in Japan.

**Table 1.** Taxonomic data from the species of the genus *Aporocotyle* in type hosts.

Species	References	Host	Locality	BL	Lateral body depression	Spine distri bution	Ratio ES/BL	Maximum extension of PC	AC/PC Ratio	LPC/BL Ratio	No. testis	Cirrus sac (length × width)	Ovary (length × width)
*A. argentinensis*	Smith, 1969	*Merluccius hubbsi*	Southwest Atlantic	4.5 (4.1–4.7)	No	AVLM	1:3.2	Posterior body end	1:2.8**	1:1.4**	41–45	520 (440–600) × 130 (110–150	250 (230–270) × 160 (140–170)
*A. australis*	Fernandez & Duran, 1985	*Merluccius australis*	Southeast Pacific	6.7 (5.4–8.0)	No	AVLM	l:3–4.4	Posterior body end**	1:3–5	1:1.4**	56–71	450 (350–530) × 130 (110–150)	170 (160–200) × 330 (280–400)
*A. garciai*	Tantalean & Martinez, 1990	*Genypterus sp.*	Southeast Pacific	3.2 (3.11–3.33)	No	LM	1: 4.4	Posterior to testes**	1:4.1	1:1.9"	96–100	280 (250–300) × 60 (50–70)	200 (180–220) × 160 (150–170)
A. garciai*	Kamegai et al., 1990	*Hoplobrotula armata*	Northwest Pacific	3.7–6.6	No	LM	1:0.9–1.4	Cirrus sac level**	1:2.8—5.1	1:1.7**	60–140	570–940 × 80–150	150–300 × 380–720
*A. keli*	Villalba & Fernandez, 1986b	*Genypterus chilensis*	Southeast Pacific	3.8 (3–0-4.7)	Yes	LM	1:3.5–4.1	Cirrus sac level***	1:2.2***	1:1.8***	35–38	360 (290–420) × 160 (120-190)	180 (160–200) × 270 (240-300)
*A. kuri*	Villalba & Fernandez, 1986b	*Genypterus maculatus*	Southeast Pacific	2.4 (1.5–3.2)	Yes	LM	1:2.9–3.8	Cirrus sac level***	1:1.8**	1:2.4**	28–32	250 (200–240) × 96 (60–120)	120 (70–150) × 190 (180–270)
*A. macfarlani*	Holmes, 1971	*Sebastodes caurinus*	Northeast Pacific	3.3–5.2	No	LM	1:3.5–	Posterior body end**	1:4.6	1:1.5’	37-63	191 (130–260) × 110 (70–150)	236 (142–340) × 161 (90–220)
*A. margolisi*	Smith, 1967	*Merluccius productus*	Northeast Pacific	4.5 (3–8–5.0)	No	AVLM	1:3.6	Posterior body end**	1:2.6**	1:1.5’	35-46	525 × 131“	280 (270–290) × 177 (150–200)
*A. nototheniae*	Parukhin, 1985	*Notothenia rossii*	Indian Ocean	10.3	No	BEC	1:11.2	Posterior body end**	1:18**	1:1.2**	135	476 × 142“	360 × 400
*A. orientalis*	Yamaguti, 1934;	*Cottunculus sp.*	Japan Sea	52–1.6	No	BEC	1:6.2	Posterior body end**	1:7.2**	1:1.2**	137–171	560 × 125	270 × 370
*A. pacifica*	Yamaguti, 1970	*Ruvettus pretiosus*	North Pacific	5.9	No	BEC	1:7.8	Posterior body end**	1:8.0**	1:12**	100	350 × 100	250 × 110
*A. simplex*	Thulin, 1980	*Platichthys flesus*	Baltic Sea	3.4–9.4	No	LM	1:3–6	Posterior body end**	1:7.8**	1:1.3**	110–203	577 × 115“	259 × 422“
*A. smithi*	Parukhin & Tkachuk, 1980	*Genypterus capensis*	Indian Ocean	4.0	No	BEC	1:4.8	Posterior end of testicular field**	1:3.5**	1:1.7**	36	340 × 130“	280 × 340“
*A. spinosicanalis*	Williams, 1958	*Merluccius merluccius*	Northeast Atlantic	4.0–6.4	No	AVLM	1:2.7	Posterior body end	1:2.1**	1:1.6**	25-35	–	400 × 210
*A. theragrae*	Ichihara, 1970	*Theragra chalcogramma*	Japan Sea	6.6–7.7	No	AVLM	1:6.0–7.7	Posterior body end**	1:8.3**	1:1.1**	98-125	380–390 × 180–240	290–-360 × 520–610
*A. wilhelmi*	Villalba & Fernandez, 1986a	*Merluccius gayi gayi*	Southeast Pacific	3.6 (2.6–5.1)	No	AVLM	1:2.6–3.6	Posterior body end	l:1.6–3.0	1:1.5**	33–40	350 (250–460) × 120 (90–170)	120 (87–160) × 220 (130–290)
*A. ymakara*	Villalba & Fernandez, 1986b	*Genypterus blacodes*	Southeast Pacific	2.1 (1.58–2.74)	No	LM	1:2.1–2.6	Cirrus sac level***	1:1.1–1.3***	1:2.5–3.3***	18–21	220 (212–228) × 61 (49–79)***	119 (93–142) × 143 (114–168)***
*A. ymakara*	Present study	*G. blacodes*	Southwest Atlantic	1.7	No	LM	1:2.6	Cirrus sac level	1:1.8	1:2.5	18	263 × 70	121 × 144
*A. mariachristinae* n. *sp.*	Present study	*G. blacodes*	Southwest Atlantic	2.7 (1.9–3.4)	No	LM	1:2.7–3.9	Posterior body end	l:2.6–4.7	1:1.5–2.3	37–39	280 (140–400) × 70 (40–80)	175 (133–237) × 155 (89–215)

Body length in millimeters, other measurements in micrometers. BL: body length; ES: esophagus; AVLM: anterior ventral body and lateral margins; LM: lateral margins; BEC: body entirely covered; AC: anterior caeca; PC: posterior caeca; LPC: long posterior caecum;

* data from *A. garciai* collected in *H. armata* are also included as it is the only *Aporocotyle* species from *Genypterus* spp. described in other host species;

** measured from the figure in the species descriptions;

*** measured from type material.

During a parasitological survey of teleosts collected from the Argentine Patagonian shelf, some specimens of *Aporocotyle* spp. were found in the bulbus arteriosus and ventral aorta of pink cusk-eels *G. blacodes*. One single specimen was identified as *A. ymakara*, previously found in the same host from the coast of Chile (Villalba & Fernández, 1986). The rest of the specimens clearly differed from all the known species of *Aporocotyle*. The aim of present study is to describe the new species of *Aporocotyle* and the specimen of *A. ymakara* from Argentina.

## Material and Methods

### Sample Collection

A total of 52 specimens of *G. blacodes* measuring from 24.7 to 92.4 cm in total length, were collected between 2007 and 2010 from two zones of the Argentinean shelf: north (42° 45’ S – 42° 59’ S, 61° 09’ W – 62° 58’ W) and central Patagonia (47° 00’ S – 47° 19’ S, 61° 59’ W – 64° 25’ W). Fish were collected by commercial bottom trawling vessels and kept fresh in ice (n = 8) or deep frozen at - 20 °C (n = 44). The heart and bulbus arteriosus of fresh and thawed fish were removed, placed in seawater and examined under a stereomicroscope. A total of 18 blood flukes were found and fixed in 70 % ethanol (n = 16) or in 100 % ethanol (n = 2). Additionally, three fresh Argentinean hakes, *Merluccius hubbsi* Marini, 1933 (total length, 38-48 cm) trawled on March 2010 from north Patagonia (42° 45’ S – 42° 59’ S, 61° 09’ W – 62° 58’ W) were examined for blood flukes. A total of 11 specimens were collected from the bulbus arteriosus, fixed in 70 % ethanol (n = 8), or in 100 % ethanol (n = 3).

### Morphological Description

15 blood flukes from 13 fish, fixed in 70 % ethanol (11 parasites from fresh fish and 4 from thawed fish), were used for morphological study, stained with iron acetocarmine (n = 13) or alum carmine (n = 2), dehydrated in ethanol, cleared in dimethyl phthalate, and mounted in Canada balsam. Specimens were examined using a compound microscope equipped with bright field and differential interference contrast optics. Morphometric measurements were taken from drawings made with the aid of a drawing tube. Measurements are reported in micrometers and presented as the mean followed in parentheses by the range and the number of structures measured. The same procedure was followed for the specimens collected from the Argentinean hakes (4 worms stained with iron acetocarmine). Blood flukes were identified as *A. argentinensis* according to Smith (1969), and were used for morphological comparison with the specimens collected from *G. blacodes*.

For morphological comparison with the species studied type specimens of the following *Aporocotyle* spp., deposited in the Zoology Museum of the University of Concepción (Concepción, Chile (ZMUC)) and the US National Parasite Collection (Beltsville, Maryland, USA (USNPC)), were examined and measured: *A. keli* – one paratype (ZMCU No. 7811); *A. kuri* – one paratype (ZMCU No. 7808); *A. wilhelmi* Villalba & Fernández, 1986 – three paratypes (USNPC No. 79462: MT24-19K, MT24-19L, and MT24-19N); and *A. ymakara* – five paratypes (ZMCU No. 7759 and USNPC No. 79463: MT24-20A, MT24-20B, MT24-20G, and MT24-20H). Additionally, 3 vouchers of *A. spinosicanalis* Williams, 1958, collected from the heart of *Merluccius merluccius* L. from the Western Mediterranean from the private collection of Dr. F. E. Montero, were studied. Measurements from previously described species were obtained from specimens or original descriptions (manuscripts or drawings), depending on the availability (see Table I).

Ecological terms follow Bush *et al.* (1997) and Rósza *et al.* (2000). The prevalence, mean abundance and mean intensity are presented as the number followed in parentheses by the 95 % confidence intervals (CI). The 95 % CI for prevalence was set with Sterne’s exact method (Reiczigel, 2003), while the 95 % CIs for the mean abundance and mean intensity were estimated with 20,000 bootstrap replications with the statistical software Quantitative Parasitology v. 3.0 (Reiczigel & Rósza, 2005).

Museums in which type specimens have been deposited, are indicated as follows: Natural History Museum, Paris, France (NHM Paris); ZMUC, Concepción, Chile. Voucher of *A. ymakara* has been deposited in NHM.

Paris. Vouchers of *A. argentinensis* have also been deposited in NHM Paris (HEL260 and HEL261) and ZMUC (N°37106 and N°37107).

### Molecular Analysis

For DNA extraction, the anterior half of each blood fluke specimen fixed in 100 % ethanol from *G. blacodes* was dissolved in 300 µl of TNES urea (10 mm Tris-HCl (pH 8), 125 mM NaCl, 10 mM ethylenediaminetetraacetic acid, 0.5 % sodium dodecyl sulphate, 4 M urea). The posterior half of each fluke was processed as previously described for morphological analysis, in order to confirm their specific status. The samples were digested with 100 µg/mL proteinase K overnight at 55 °C. DNA was extracted using a conventional phenol–chloroform protocol following Alama-Bermejo *et al.* (2011). The extracted DNA was resuspended in 30 µL of RNAse/DNAse free water and left to dissolve overnight in the fridge. Polymerase chain reactions (PCR) were performed with a programmable thermal cycler (Techne, TC-512, GMI) in a final volume of 30 µL containing 0.5 U of Thermoprime Plus DNA polymerase and 3 µL of the related 10× buffer with 1.5 mM MgCl_2_ (ABgene), 0.2 mM of each dNTP, 0.5 µM of each primer and approximately 100 ng of template DNA.

Partial 18S rDNA and 28S rDNA were amplified from the genomic ribosomal DNA (rDNA) gene. The primers used in the PCR reaction were the forward primer WormA (5’-GCGAATGGCTCATTAAATCAG-3’) and the reverse primer WormB (5’-CTTGTTACGACTTTTACTTCC- 3’) (Littlewood & Olson, 2001) for 18S rDNA; the forward primer U178 (5’-GCACCCGCTAAYT-TAAG- 3’) and the reverse primer L1642 (5’-CCAGCGCCATCCATTTTCA- 3’) (Lockyer *et al.*, 2003) for 28S rDNA.

The PCR protocol was conducted as follows: denaturation (95 °C for 3 min) followed by 40 cycles of amplification. Cycling consisted of 95 °C for 30 s, 55 °C as annealing temperature for 30 s and 72 °C for 2 min. Final extension was allowed at 72 °C for 7 min. After checking for the presence of PCR amplicons in a 1 % agarose gel in sodium acetate buffer, the PCR products were purified for sequencing using the GFX PCR DNA and Gel Band Purification Kit (GE Healthcare UK Ltd.). PCR primers and additional primers Lin3 (5’-GCGGTAATTCCAGCTCCA-3’) (Lin *et al.*, 1999) and LSU1200R (5’-GCATAGTTCACCATCTTTCGG-3’) (Lockyer *et al.*, 2003) were used for sequencing of the 18S and 28S rDNA fragments respectively. Cycle sequencing was conducted in a 48 capillary ABI 3730 sequencer (Applied Biosystems) using the BIG Dye Terminator v 3.1 Ready Sequencing Kit (Applied Biosystems) according to the manufacturer’s instructions. Sequences obtained were submitted to the Basic Local Alignment Search Tool (BLAST) on GenBank^TM^ to identify the closest relatives published to date. The same protocols were used to obtain the partial 28S rDNA sequence of the whole specimen of *A. argentinensis* from *M. hubbsi* fixed in 100 % ethanol.

## Results

### *Aporocotyle Mariachristinae* n. sp. (Figs 1–6, 11)

Type host: pink cusk-eel, *Genypterus blacodes* (Forster, 1801) (Ophidiiformes: Ophidiidae). Host size: total length, 30.6-92.4 cm.

Site in host: bulbus arteriosus and ventral aorta.

Type locality: north Patagonia (42° 45’ S – 42° 59’ S, 61° 09’ W – 62° 58’ W); and central Patagonia (42° 2’ S – 47° 19’ S, 61° 59’ W – 64° 25’ W), Argentina.

Type specimens: holotype (NHM Paris HEL255), three paratypes (NHM Paris HEL256, HEL257 and HEL258), three paratypes (ZMUC N°37103, N°37104 and N°37105).

Infection parameters: prevalence, 25.0 % (14.9-38.3); abundance, 33.0 (17.0-63.0); intensity, 1.31 (1.0-1.9).

Sequence data: GenBank Accession number JX094801 (*Aporocotyle mariachristinae* partial 18S rDNA sequence, ex. *Genypterus blacodes*); JX094802 (*Aporocotyle mariachristinae* partial 28S rDNA sequence, ex. *Genypterus blacodes*).

Etymology: the species is named in memory of María Cristina Orts Pino, mother of the first author.

**Figs 1, 2.**
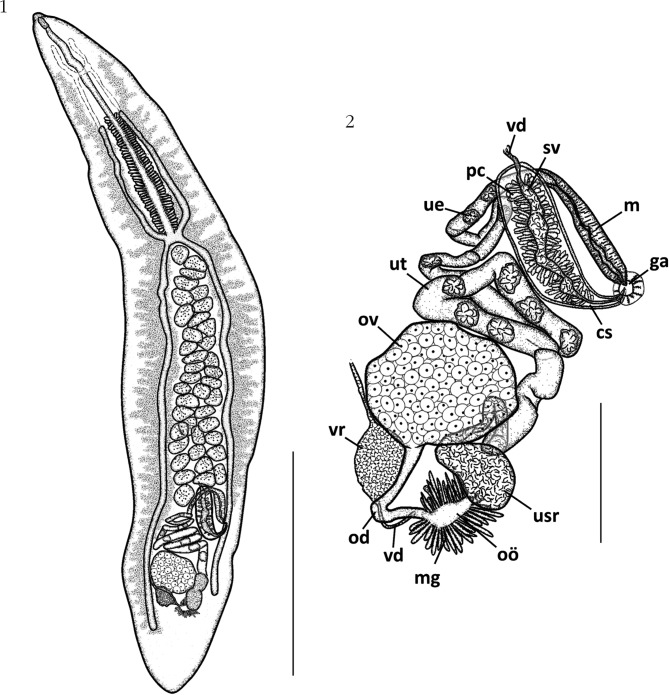
*Aporocotyle mariachristinae* n. sp. from *Genypterus blacodes*: 1, whole worm, holotype, ventral view; 2, reproductive organs of the holotype, ventral view. Scale bars: Fig. 1: 1,000 µm; Fig. 2: 200 µm. Abbreviations: vd, vas deferens; sv, seminal vesicle; pc, prostatic cells; ue, uterine egg; m, metraterm; ut, uterus; ga, genital atrium; ov, ovary; cs, cirrus sac; vr, vitelline reservoir; usr, uterine seminal receptacle; od, oviduct; vd, vitelloduct; oö, oötype; mg, Mehlis’ gland.

**Figs 3–6.**
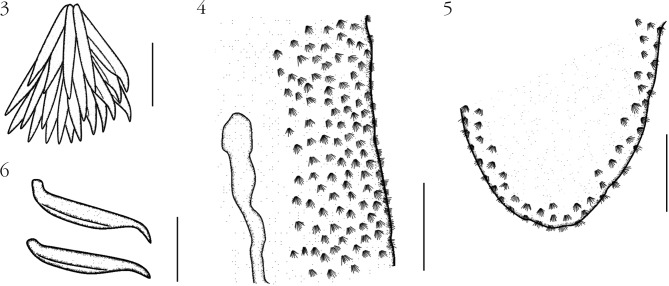
*Aporocotyle mariachristinae* n. sp. from *Genypterus blacodes*: 3, cluster of spines; 4, distribution of clusters of spines along ventro-lateral body margins near the anterior left caecum, ventral view; 5, distribution of clusters of spines at ventral posterior body end; 6, tegumental spines. Scale bars: Figs 3 and 4: 100 µm; Figs 5 and 6: 10 µm.

#### • Description

Based on 14 mounted specimens. Aporocotylidae, *Aporocotyle* Odhner, 1900. Body lanceolate, pointed at anterior end and blunt at posterior end (Figs 1, 11), 2,730 (1,910-3,380, n = 12) long and 450 (220-670, n = 12) wide. Maximum body width approximately at mid-level. Tegument bearing clusters of approximately 20 spines each (Fig. 3) in ventro-lateral fields, slightly extending dorsally. Spines approximately 20 long (Fig. 6). Ventrally, spines are distributed along entire body length, never joining at sagittal axis (Fig. 11), but reaching closer at anterior half body, occupying approximately 2/3 distance between body margin to caecum (Figs 4 & 11). Spines less abundant at posterior half body (Figs 5 & 11). Oral sucker absent. Mouth ventral, leading to a buccal capsule 55 (47-63, n = 4) long and 24 (18-31, n = 4) wide. Esophagus 831 (613-1,024, n = 8) long or 31 % (26-37 %) of body length, surrounded by glands approximately from its midlevel (anterior to level of anterior caeca distal end) to caeca bifurcation. Intestine X- or H-shaped (Figs 1 & 11). Anterior caeca almost equal in length. Anterior caeca/body length ratio: 1:6.5 (1:4.8-7.6). Right anterior caecum 435 (342-598, n = 8); left anterior caecum 418 (289-646, n = 8). Posterior caeca unequal: right posterior caecum always longer than left posterior caecum. Right posterior caecum 1,462 (1,061-1,877, n = 9), about 54 % (44- 67 %) of body length, ending from mid-level of ovary to near posterior body end (at mid-level of ovary (n = 3) (Fig. 11), posterior to reproductive organs (n = 9)

(Fig. 1), or near to posterior body end (n = 2)). Left posterior caecum 1,345 (974-1,772, n = 9), ending from mid-level of cirrus sac to posterior to reproductive organs (at mid-level of cirrus sac (n = 6), at proximal female genitalia level (n = 5) (Figs 1, 11), or posterior to reproductive organs (n = 3)). Anterior caeca/right posterior caecum length ratio 1:3.5 (1:2.7-5.1).

Testes irregularly-shaped (Fig. 1), 37-39 (n = 4) in number, 70 (40-125, n = 49) long axis and 81 (24-118, n = 49) wide axis. Testes intercaecal, between caecal bifurcation and anterior to reproductive organs. Vas deferens extending from posterior border of testicular field. Cirrus sac claviform, 271 (144-396, n = 7) long and 70 (41-84, n = 10) wide, with thin muscular wall, directing sinistrally, ending at genital atrium (Fig. 2). Seminal vesicle elongate, slightly sinuous, in middle of cirrus sac, surrounded by prostatic cells (Fig. 2). Cirrus not observed. Genital atrium and genital pore sinistral, dorsal to left posterior caecum, at about 75 % (64- 84 %) of body length from anterior body end. Ovary central or slightly dextral (Figs 1, 11), sub-ellipsoidal, in posterior 1/8 of body, 175 (133-237, n = 10) long and 160 (89-215, n = 10) wide (Fig. 2). Oviduct running posteriorly from posterior margin of ovary and turning sinistrality (Fig. 2). Oötype surrounded by Mehlis’ gland, connecting to uterine seminal receptacle. Laurers’ canal absent. Uterus intercaecal, extending from uterine seminal receptacle and coiling from ovary to level of posterior testes, curving dorso-sinistrally (Fig. 2). Metraterm muscular 174 (117-238, n = 4) long, parallel to cirrus sac (Fig. 2). Vitelline follicles small, compact. Vitelline fields branched, extending anteriorly from nerve commissure to posterior end of right posterior caecum on dextral side, and anterior to genital atrium on sinistral side. Vitelline reservoir posterior to ovary. Vitelloduct connects to oviduct at sagittal axis (Fig. 2). Eggs thin-shelled, irregular to ellipsoidal shaped 35 (30-43, n = 25) long and 34 (25- 43, n = 25) wide.

Nerve commissure ventral, approximately at first quarter of esophagus (Fig. 1). Nerve cords extending anteriorly from approximately 1/8 of the esophagus; indistinct posteriorly. Excretory pore not observed.

#### • Comparison

Among the 16 known species of *Aporocotyle*, *A. mariachristinae* is distinguished from the other species by its unique asymmetric arrangement of posterior caeca. This is the only species known of the genus with the right posterior caecum ending at the area between mid-level of ovary and posterior body end, and the left posterior caecum, always shorter, ending at the area between mid-level of cirrus sac and posterior to reproductive organs, never ending near posterior body end. Posterior caeca reach near to the posterior body end in most of the species of *Aporocotyle*, except for those in fishes of the Ophidiiformes (*Genypterus* spp. and *H. armata*, see Table I) in which posterior caeca never reach to ovary (Parukhin & Tkachuk, 1980; Villalba & Fernández, 1986; Tantaleán & Martínez, 1990; Kamegai *et al.*, 2002). *A. mariachristinae* would be the first species in an ophidiiform fish in which at least one posterior caecum extends beyond the anterior ovary end. The new species clearly differs from *A. ymakara*, the other species described from *G. blacodes*, by the higher number of testes (37-39 instead of 18-21), the absence of distal curves at caeca (see Fig. 1 in Villalba & Fernández, 1986), the different esophagus/body length ratio (1:2.7-3.9 instead of 1:2.1- 2.6), and the different anterior caeca/posterior caeca length ratio (1:2.6-4.7 instead of 1:1.1-1.3). Additionally, in *A. ymakara*, the left posterior caecum can be equal or longer than the right posterior caecum (see Villalba & Fernández, 1986), while in the new species is always shorter. The most similar species is *A. keli*, described from *G. chilensis* from the Southeast Pacific, with similar size and number of testes. This species differs from *A. mariachristinae* by the presence of a “muscular fold at posterior body region at level of genital atrium” (Villalba & Fernández, 1986, see discussion for comments on this trait) together with the different host and the different posterior caeca arrangement.

*Aporocotyle mariachristinae* also differ from *A. argentinensis*, *A. australis*, *A. margolisi*, *A. nototheniae*, *A. orientalis*, *A. pacifica*, *A. smithi*, *A. spinosicanalis*, *A. theragrae*, and *A. wilhelmi*, in which the clusters of spines can join ventrally at sagittal axis (Fig. 10). Other specific differences with the other *Aporocotyle* species are reported in Table I.

#### • Molecular characterization

Partial 18S rDNA sequences were obtained from *A. mariachristinae* (1,726 bp) and sequence submission to the BLAST server showed that the 18S rDNA closest sequence matches were *A. spinosicanalis* (GenBank Accession Number AJ287477; Query coverage 100 %; Maximun identity 97 %). Partial 28S rDNA sequences from *A. mariachristinae* (1,554 bp) submitted to the BLAST server showed that the 28S rDNA closest sequences matches were also *A. spinosicanalis* (Gen- Bank Accession Number AY222177; Query coverage 81 %; Maximun identity 94 %).

For molecular comparison, partial 28S rDNA sequence was also obtained from *A. argentinensis* (1,540 bp) (GenBank Accession number JX094803; *A. argentinensis* partial 28S rDNA sequence, ex. *M. hubbsi*). The alignment of the partial 28S rDNA sequences of *A. mariachristinae* and *A. argentinensis* with the only sequence of the genus available in the database, *A. spinosicanalis*, showed the following interspecific sequence similarities over an alignment of 1,651 bp: *A. mariachristinae*/*A. argentinensis*, 94.3 %; *A. mariachristinae*/ *A. spinosicanalis*, 93.3 %; *A. argentinensis*/ *A. spinosicanalis*, 96.8 %.

### Aporocotyle Ymakara

**Figs 7–9.**
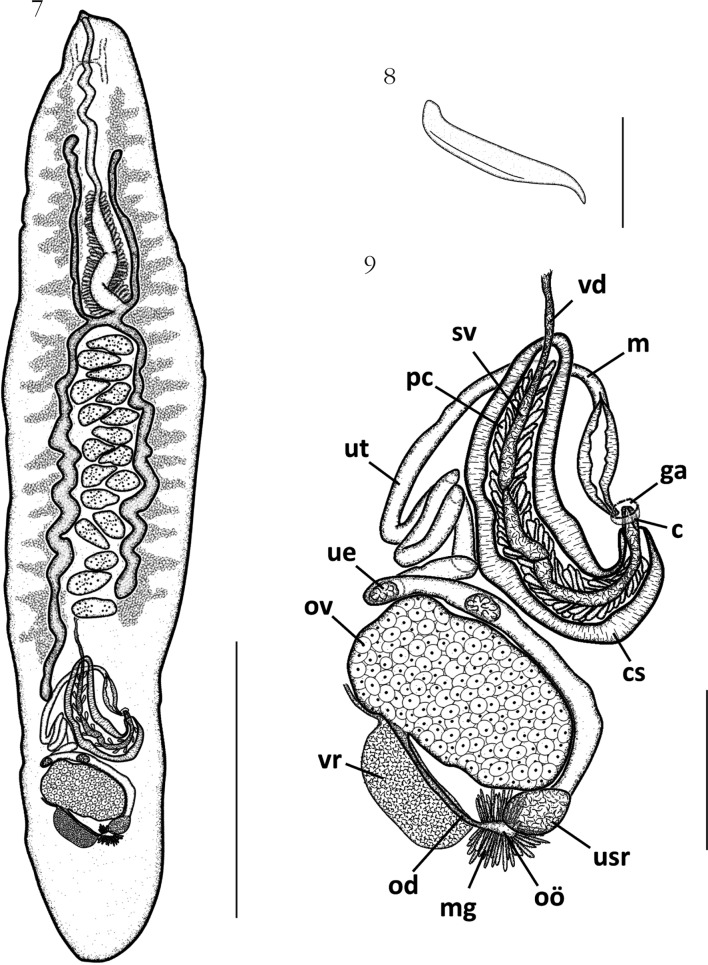
*Aporocotyle ymakara* from *Genypterus blacodes* from Patagonia, Argentina: 7, whole worm, voucher specimen, ventral view; 8, tegumental spine; 9, reproductive organs, ventral view. Scale bars: Fig. 7: 500 µm; Fig. 8: 10 µm; Fig. 9: 100 µm. Abbreviations: vd, vas deferens; sv, seminal vesicle; m, metraterm; pc, prostatic cells; ut, uterus; ga, genital atrium; c, cirrus; ue, uterine egg; ov, ovary; cs, cirrus sac; vr, vitelline reservoir; usr, uterine seminal receptacle; od, oviduct; oö, oötype; mg, Mehlis’ gland.

VILLALBA & FERNÁNDEZ, 1986 (Figs 7–9)

Type host: pink cusk-eel, *Genypterus blacodes* (Forster, 1801) (Ophidiiformes: Ophidiidae).

Host size: total length, 51.2 cm.

Type locality: Arauco Gulf (37° 00’ S – 73° 20’ W), Chile.

New locality: central Patagonia (42° 2’ S, 47° 19’ S – 61° 59’ W, 64° 25’ W), Argentina.

Voucher specimen: NHM Paris (HEL259).

Infection parameters: prevalence, 1.9 (0.1-10.2); abundance, 2.0 (0.0-6.0); intensity, 1.0.

#### • Description of the specimen from Patagonia, Argentina

Measurements from the single specimen collected in the present study. Aporocotylidae, *Aporocotyle* Odhner, 1900, *A. ymakara* Villalba & Fernández, 1986. Body lanceolated (Fig. 7), 1,698 long and 364 wide. Maximum body width approximately at mid-level. Tegument bearing clusters of approximately 20 spines each, in ventro-lateral fields, slightly extending dorsally. Spines approximately 18 long (Fig. 8). Ventrally, spines are distributed along entire body length, never joining at sagittal axis, but reaching closer at anterior half body, occupying, approximately 2/3 distance between body margin to caecum (see similar distribution of paratype in Fig. 12). Spines less abundant at posterior half body. Mouth subterminal. Buccal capsule not observed. Esophagus 659 long (39 % of body length), surrounded by glands at its posterior half (posterior to level of anterior caeca distal end) until caeca bifurcation. Intestine H-shaped. Right anterior caecum slightly longer than the left anterior caecum. Anterior caeca/body length ratio 1:5.3. Right anterior caecum 333; left anterior caecum 310. Posterior caeca unequal: right posterior caecum longer than left posterior caecum. Right posterior caecum 674 (about 40 % of body length), ending approximately at cirrus sac level (Fig. 7). Left posterior caecum 508, ending before the posterior end of testes field (Fig. 7). Anterior caeca/right posterior caecum length ratio 1:2.1.

**Figs 10–12.**
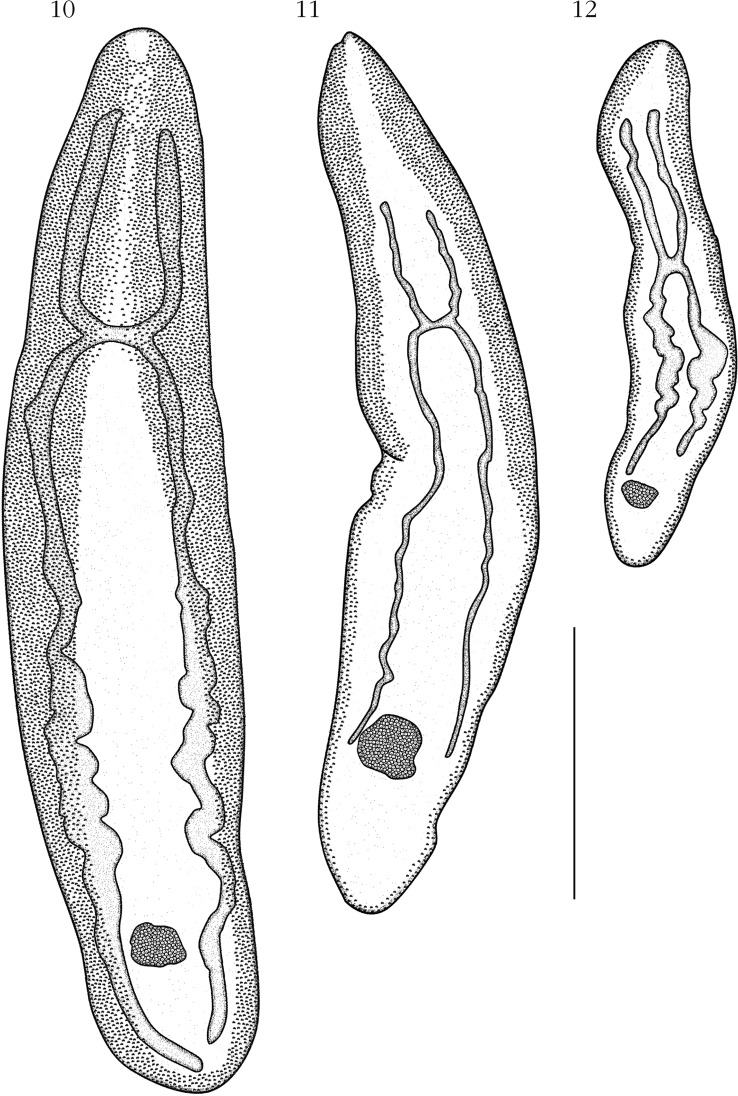
Extension of caeca and diagrammatic representation of distribution of clusters of spines along the entire body length in three species of *Aporocotyle*, ventral view: 10, *A. argentinensis*, voucher; 11, *A. mariachristinae* n. sp., paratype; 12, *A. ymakara*, paratype (ZMCU No. 7759). Scale bar: 1,000 µm.

Testes irregularly-shaped (Fig. 7), 18 in number, 36 (23-64, n = 18) long axis and 65 (39-81, n = 18) wide axis. Testes intercaecal, between caecal bifurcation and anterior to reproductive organs. Vas deferens extending from posterior border of testicular field. Cirrus sac claviform, 263 long and 70 wide, with a thick muscular wall, directing sinistrally, ending at genital atrium (Fig. 9). Seminal vesicle elongate, slightly sinuous, in middle of cirrus sac, surrounded by prostatic cells (Fig. 9). Cirrus short, 15 long (Fig. 9). Genital pore dorsosinistral, at 78 % of body length from anterior body end. Ovary central, slightly dextral, sub-ellipsoidal, in posterior 1/8 of body, 121 long and 144 wide (Fig. 9). Oviduct running posteriorly from posterior margin of ovary, sinistrally oriented (Fig. 9). Oötype surrounded by Mehlis’ gland, connecting to uterine seminal receptacle. Laurers’ canal absent. Uterus extending from uterine seminal receptacle and coiling from posterior ovary end to level of anterior cirrus sac end, curving dorso-sinistrally (Fig. 9). Metraterm muscular 79 long, parallel to cirrus sac (Fig. 9). Vitelline follicles small, compact. Vitelline fields branched, extending, on dextral side, from nerve commissure to posterior end of right posterior caecum, and, on sinistral side, slightly beyond posterior end of left posterior caecum. Vitelline reservoir posterior to ovary (Fig. 9). Vitelloduct after vitelline reservoir short, connecting dorsally to oviduct. Eggs thin-shelled, irregular to ellipsoidal shaped 16 (13-19, n = 2) long and 20 (17-23, n = 2) wide.

Nerve commissure ventral, approximately at first quarter of esophagus (Fig. 7). Nerve cords indistinct. Excretory pore not observed.

#### • Remarks

The morphological traits of the specimen collected from Argentina coincide with those described by Villalba & Fernández (1986b) for *A. ymakara* from Chile: body size, esophagus/body length ratio, number of testes, and distribution of spines along the body (see Table I). The extension of the caeca showed a slight variability in specimens of both localities. Right posterior caecum ends before cirrus sac (three paratypes), or at cirrus sac level (two paratypes and the specimen from Argentina). Left posterior caecum ends at last testis level in all specimens (Chilean and Argentinean). Villalba & Fernández (1986b) reported that the distal end of caeca of some specimens was curved, however this curvature was not observed in the paratypes or in the Argentine specimen analysed in present study. The ratio between anterior caeca length/posterior caeca length of *A. ymakara* was 1:2.8-3.5 according to Villalba & Fernández (1986b), however revision of paratypes from Chile revealed that the ratio was 1:1.1-1.3.

## Discussion

The most used morphological traits to differentiate species of the genus *Aporocotyle* are body shape, distribution of spines along the body, esophagus/body length ratio, anterior caeca/posterior caeca length ratio, number of testes, shape of cirrus sac, shape of ovary, presence of a “muscular fold” at level of genital atrium, and extension of posterior caeca (Smith, 1967, 1969; Holmes, 1971; Parukhin & Tkachuk, 1980; Villalba & Fernández 1986b).

The distribution of spines along the body has been commonly used to separate species of *Aporocotyle* (see Smith, 1967, 1969; Ichihara, 1970; Holmes, 1971). From the species examined in this study, two different patterns of distribution of spines have been observed: (*i*) in *A. argentinensis*, *A. spinosicanalis*, and *A. wilhelmi*, the cluster of spines are arranged along the ventromarginal areas of the body joining at sagittal axis from posterior margin of mouth to caeca bifurcation (Fig. 10); and (*ii*) in specimens of *A. keli*, *A. mariachristinae*, and *A. ymakara*, spines are arranged along the ventro-marginal area of the body, never joining at sagittal axis (Figs 11, 12). The spine arrangement of the paratype of *A. kuri* was in poor conditions and was unable to be described. The first type of spine arrangement is similar to those of *A. argentinensis*, *A. australis*, *A. margolisi*, *A. simplex*, *A. spinosicanalis*, *A. theragrae*, and *A. wilhelmi* (Williams, 1958; Smith, 1967, 1969; Ichihara, 1970; Thulin, 1980; Fernández & Durán, 1985; Villalba & Fernández, 1986a), while the second type of spine arrangement is similar to those described for *A. garciai*, *A. keli*, *A. kuri*, *A. macfarlani*, and *A. ymakara* (Holmes, 1971; Villalba & Fernández, 1986; Tantaleán & Martínez, 1990). A third pattern of distribution of spines has been described in *A. orientalis*, *A. pacifica*, *A. smithi*, and *A. nototheniae* where the full body surface is covered with spines (Yamaguti, 1934, 1970; Parukhin & Tkachuk, 1980; Parukhin, 1985). However, this trait should be revised as the description of the distribution of spines in these species is not sufficiently detailed.

The presence of a “muscular fold” was proposed by Villalba & Fernández (1986b) as a morphological character to differentiate *A. kuri* and *A. keli* from other species. These authors described this structure as “a muscular fold at posterior body region at level of genital atrium” (“*un repliegue muscular en la región posterior a nivel del atrio genital*”). Villalba & Fernández (1986b) also reported that in these species, due to this fold, the region posterior to genital atrium was sinistrally bent, oblique to perpendicular to longitudinal body axis. The authors did not provide more information or drawings on what kind of musculature existed within this fold. In Figures 2 and 3 of Villalba & Fernández (1986b) (*A. kuri* and *A. keli*, respectively) a marked lateral body depression at level of genital atrium can only be noticed. Thereafter, Tantaleán & Martínez (1990) again referred to the absence of this trait to describe *A. garciai*. Moreover, Kamegai *et al.* (2002) mistranslated the trait “repliegue muscular” as “muscular sphincter at female pore” in their description of new specimens of *A. garciai* from Japan. We did not observed a body depression or a muscular fold at level of genital atrium, or a curvature of the posterior region in the paratypes of *A. kuri* and *A. keli* examined in this study. Therefore this character does not appear to be recommendable for these species’ diagnosis.

Species of *Aporocotyle* have been classified in two groups according to the extension of posterior caeca (Villalba & Fernández, 1986): (*i*) species of *Aporocoty le* described from fishes of the orders Gadiformes, Perciformes, Pleuronectiformes and Scorpaeniformes which have posterior caeca ending near the posterior body end (Fig. 10) (Yamaguti, 1934, 1970; Williams, 1958; Smith, 1967, 1969; Ichihara, 1970; Holmes, 1971; Thulin, 1980; Fernández & Durán, 1985; Parukhin, 1985; Villalba & Fernández, 1986a); and (*ii*) species of *Aporocotyle* described from ophidiid fishes, with posterior caeca never ending near the posterior body end and never reaching to the level of the ovary (Fig. 12) (Parukhin & Tkachuk, 1980; Villalba & Fernández, 1986; Tantaleán & Martínez, 1990). According to this classification, *A. mariachristinae* could be considered to belong to a third intermediate group with at least one posterior caecum reaching to the area from mid-level of ovary to posterior body end (Fig. 11). Unfortunately, the existence of different phylogenetic groups of *Aporocotyle* species could not be confirmed in present study with molecular analyses as only three sequences exist (two of them provided in this study). We encourage authors to provide more molecular data on aporocotylid genera to explore phylogenetic relationships.

Currently, there are six species of *Aporocotyle* described from four species of fishes from the genus Genypterus (see Parukhin & Tkachuk, 1980; Villalba & Fernández, 1986b; Tantaleán & Martínez, 1990; present study). To our knowledge, this is the only fish genus in which two different species of *Aporocotyle* infect the same host species. Moreover, *A. mariachristinae* and *A. ymakara* could not be the only two *Aporocotyle* species infecting the same host species: although the specific identity of the definitive host for *A. garciai* is unknown (reported as *Genypterus* sp. in Tantaleán & Martínez, 1990), *G. chilensis* or *G. maculatus* must be the type host as they are the only species of *Genypterus* distributed along the Peruvian coast (Nielsen *et al.*, 1999), and both fish species harbour other *Aporocotyle* species (*A. keli* and *A. kuri* respectively, see Table I). More than one blood fluke species of the same genus in the same host have been previously reported: *i.e.*, *Cardicola* Short, 1953 (see Nolan & Cribb, 2006) and *Paradeontacylix* McIntosh, 1934 (see Ogawa & Egusa, 1986; Repullés-Albelda *et al.*, 2008).
